# Short-chain-fatty acid valerate reduces voluntary alcohol intake in male mice

**DOI:** 10.21203/rs.3.rs-3496323/v1

**Published:** 2023-10-30

**Authors:** Suresh C Bokoliya, Jordan Russell, Yair Dorsett, Hunter Panier, Vijender Singh, Lauren Daddi, Hanshu Yuan, Liv R. Dedon, Zhongmao Liu, Jessica R. Barson, Jonathan Covault, Jason A. Bubier, Yanjiao Zhou

**Affiliations:** University of Connecticut Health Center; University of Connecticut Health Center; University of Connecticut Health Center; University of Connecticut Health Center; University of Connecticut Health Center; University of Connecticut Health Center; University of Connecticut Health Center; University of Connecticut Health Center; University of Connecticut; Drexel University College of Medicine; University of Connecticut Health Center; Jackson Laboratory; University of Connecticut Health Center

**Keywords:** Alcohol drinking, SCFA, Sodium valerate, GABA, Microbiome

## Abstract

**Background:**

Despite serious health and social consequences, effective intervention strategies for habitual alcohol binge drinking are lacking. Development of novel therapeutic and preventative approaches is highly desirable. Accumulating evidence in the past several years has established associations between the gut microbiome and microbial metabolites with drinking behavior, but druggable targets and their underlying mechanism of action are understudied.

**Results:**

Here, using a drink-in-the-dark mouse model, we identified a microbiome metabolite-based novel treatment (sodium valerate) that can reduce excessive alcohol drinking. Sodium valerate is a sodium salt of valeric acidshort-chain-fatty-acid with similar structure as γ-aminobutyric acid (GABA). Ten days of oral sodium valerate supplementation attenuates excessive alcohol drinking by 40%, reduces blood ethanol concentration by 53%, and improves anxiety-like or approach-avoidance behavior in male mice, without affecting overall food and water intake. Mechanistically, sodium valerate supplementation increases GABA levels across stool, blood, and amygdala. It also significantly increases H4 acetylation in the amygdala of mice. Transcriptomics analysis of the amygdala revealed that sodium valerate supplementation led to changes in gene expression associated with functional pathways including potassium voltage-gated channels, inflammation, glutamate degradation, L-DOPA degradation, and psychological behaviors. 16S microbiome profiling showed that sodium valerate supplementation shifts the gut microbiome composition and decreases microbiome-derived neuroactive compounds through GABA degradation in the gut microbiome.

**Conclusion:**

Our findings suggest that the sodium valerate holds promise as an innovative therapeutic avenue for the reduction of habitual binge drinking, potentially through multifaceted mechanisms.

## Background

Binge drinking is the most common form of excessive alcohol consumption in the US. It is characterized by frequent and intense drinking episodes [1]. Excessive alcohol use leads to significant health and social problems as well as a substantial economic cost [2, 3]. In addition, elevated anxiety and depression during alcohol withdrawal act as strong negative forces that drive individuals towards compulsive drug-seeking behavior and increase the risk of relapse [4]. Despite of three existing medications, in the past two decade, no new drugs for alcohol use disorder have been approved by US FDA. Development of pharmacological interventions to reduce alcohol drinking remains a high priority for the mission of National Institute on Alcohol Abuse and Alcoholism [5].

Recent advances in the microbiome and alcohol research have shown that alcohol consumption can influence [6] or be influenced by the gut microbiome [7]. Acute or chronic alcohol consumption breaks down the homeostasis of gut microbiota, resulting in an overgrowth of harmful bacteria and a reduction in beneficial bacteria [8–10]. Dysbiosis of gut microbiota has been highlighted in the progression of alcoholic liver diseases [11]. Alcohol cessation showed recovery of the gut microbiota and alleviated some of the adverse effects of alcohol consumption [12]. In addition to microbiome composition, alcohol consumption causes a reduction in the production of short-chain fatty acids (SCFAs)-the primary gut microbial metabolites derived from fermentation of non-digestible carbohydrates. Alcohol consumption directly decreases the abundance of SCFA-producing bacteria [13, 14], and lowers SCFA levels in stools and blood [15, 16]. SCFAs are vital in regulating immune responses and maintaining gut and blood-brain barrier integrity [17]. SCFAs have also been shown to affect behavior, including those involved in reward, stress [18], and substance use diseases [19]. In rats, administration of SCFAs, like sodium butyrate, reduced alcohol-induced liver damage and inflammation [20]. Systemic use of sodium butyrate even produced antidepressant-like behavior in rats [21]. In contrast, acetate, another SCFA, encourages ongoing heavy drinking [22]. The underlying mechanisms by which SCFAs affect alcohol drinking is not fully understood. Modulation of epigenetic and gene expression in the brain, potentially mediated by SCFAs, may contribute to their effects on alcohol drinking behavior [23, 24].

Using a binge drinking murine model, our study identified that valeric acid, one type of SCFA with a similar structure features of the neurotransmitter γ-aminobutyric acid (GABA) [25], significantly attenuates binge drinking and reduces anxiety-like behavior. We identified multiple potential mechanisms of action of valeric acid involving epigenetic modulation, GABA regulation and microbiome reconstruction. These findings pave the way for exploring the development of a microbiome metabolite-associated therapy for excessive alcohol drinking and alcohol use disorders.

## Methods

### Animals

Male C57BL/6J mice, (RRID: IMSR_JAX:000664), aged 6–8 weeks, were obtained from Jackson Laboratories (ME, U.S.A) for the study. The mice were housed individually under a reversed 12-hour light/dark cycle, with lights off from 7:00 pm to 7:00 am. A minimum acclimation period of 2 weeks was provided for all animals before they were randomly assigned to their respective groups. Mice had free access to food (Irradiated chow, Teklad global 18% protein rodent diet, 2918, Inotiv, WI, U.S.A) and deionized water to acclimate to the testing environment. Body weight and daily food and fluid intake were monitored and noted for all animal experiments.

### Antibiotics treatment

An antibiotic cocktail (Abx) comprised of 3.0 mg/mL vancomycin (Thermo Fisher Scientific, CA, USA), 6.0 mg/mL metronidazole (Sigma, MO, U.S.A), 6.0 mg/mL ampicillin (Sigma, MO, U.S.A), and 6.0 mg/mL neomycin (Janssen Pharmaceuticalaan, Belgium) was given to mice via oral-gastric gavage for five consecutive days (250 μL Abx/gavage/mouse). In contrast, control mice were given phosphate-buffered saline (PBS; Thermo Fisher Scientific, CA, USA) as a control via oral-gastric gavage for five consecutive days, with each mouse receiving 250 μL of PBS per gavage.

### DID paradigm for binge-like ethanol drinking and SCFA administration

A standard 4-day drinking in the dark (DID) model was used to evaluate binge-like ethanol drinking patterns in mice. A 20% v/v ethanol solution was prepared by diluting 190-proof ethyl alcohol (Sigma, MO, U.S.A) with deionized water. During days 1–3, the water bottles were temporarily removed during the dark cycle of the mice, and the mice were provided with a tube containing the 20% v/v ethanol solution for a duration of 2 hours. On day four, the assessment of binge-like ethanol consumption took place, where the mice were given access to the 20% v/v ethanol solution for a period of four hours. The volume of fluid in the tube (read to the closest 100 μL) was measured immediately upon placement, and at two hours and four hours into the test. Following the completion of the four hours test, approximately 100 μL of blood was collected through cardiac puncture after CO_2_ euthanasia. Blood samples were subjected to centrifugation at 10,000 rpm for 10 minutes. The determination of blood ethanol concentrations (BEC) was performed using an Analox AM1 Analyzer (Analox Instruments, MA, U.S.A). The quantity of ethanol consumed was then calculated as grams per kilogram of the mouse’s body weight.

All SCFA were used in sodium salt form. Briefly, sodium acetate (Sigma, MO, U.S.A), sodium butyrate (Sigma, MO, U.S.A), sodium valerate (Ambeed, IL, U.S.A), and sodium chloride (NaCl; Sigma, Mo, U.S.A) were prepared at 200 mM concentration freshly in drinking water every two days. These concentrations were chosen based on earlier studies conducted in mice [26, 27]. All SCFA and NaCl solutions were pH verified to control for acidity effects on daily consumption. SCFAs were given to mice via oral drinking for 10 consecutive days before any experiment, and this regimen was maintained throughout the entire experiment.

### SCFA measurement

The fecal samples (50–100 mg) were mixed with 1 mL of a 0.5% phosphoric acid solution (Sigma, MO, U.S.A). After collection, the samples were immediately frozen at −20°C. Upon thawing, the fecal content suspensions were thoroughly homogenized by vortexing for 2 minutes and then centrifuged at 18,000×g for 10 minutes. The resulting supernatant was extracted with ethyl acetate (Sigma, MO, U.S.A) and then centrifuged in glass tubes at 3,000×g for 10 minutes. The separated organic phase was analyzed using gas chromatography with a flame ionization detector (Shimadzu GC-QP2010 SE, Tokyo, Japan). Identification of SCFAs was achieved by comparison to the chemical standards (acetic acid, propionate acid, butyric acid, isovaleric acid, and valeric acid) (Sigma, MO, U.S.A).

### Open field activity test

The open field activity test was conducted to assess the impact of sodium valerate supplementation on exploratory and anxiety-like behavior. This test is a widely used method for analyzing locomotor ability and anxiety-related emotional behaviors in C57BL/6 mice. The open field activity chamber, constructed from white Plexiglass and illuminated with daylight, had dimensions of 37.5 cm height × 40 cm length × 40 cm width. General locomotor activity was recorded for 10 min by an overhead camera and analyzed using an automated video tracking system (ANY-maze v.4.6, Stoelting, IL, U.S.A, RRID:SCR_014289). The primary variables of interest included the total distance traveled (cm), the number of entries in the center area (20 cm length × 20 cm width), and the percentage of time spent in the center area of the open field.

### Measurement of GABA

GABA levels in plasma, stool, and amygdala samples were determined using an ELISA kit (LDN, Labor Diagnostika Nord, Nordhorn, Germany) following the manufacturer’s instructions. Plasma samples were used directly without any additional preparation. For stool and amygdala, samples, they were first thawed and then homogenized in a solution consisting of 0.01N HCl (Thermo Fisher Scientific, MA, U.S.A), 1mM EDTA (Thermo Fisher Scientific, MA, U.S.A), and 4mM sodium metabisulfite (Thermo Fisher Scientific, MA, U.S.A). After homogenization, the samples underwent centrifugation at 3000×g for 5 minutes at 4°C before GABA estimation. To summarize the assay procedure, plasma, homogenized stool, and amygdala samples, and the kit’s standards were all processed on an extraction plate. They were subsequently derivatized using an equalizing reagent and subjected to a standard competitive ELISA conducted in GABA-coated 96-well microtiter strips. The optical density (OD) of the solution within the wells was rapidly read at 450 nm using a 96-well plate reader (iMark^™^ Microplate Absorbance Reader; Biorad, CA, U.S.A, RRID:SCR_023799). The OD data was employed to determine the GABA concentration via a standard curve.

### Measurement of histone acetylation in amygdala

Amygdala samples were thawed, and the histone fractions were prepared using a commercial histone extraction kit (Abcam, Cambridge, UK) following manufacturers instruction. The bulk acetylation of histone H3 and H4 was determined using commercial ELISA kits according to the manufacturer’s instructions (Abcam, Cambridge, UK). The OD of the samples was measured using a 96 well plate reader at 450 nm (iMark^™^ Microplate Absorbance Reader; Biorad, CA, USA). The amounts of acetyl histone H3 and H4 were measured by comparing to the kit supplied standards.

### RNASeq of amygdala and data analysis

Total RNA was isolated from amygdala samples using the Direct-zol RNA Microprep Kit (Zymoresearch, CA, U.S.A). Quantification of total RNA and assessment of its purity ratios were performed using the NanoDrop 2000 spectrophotometer (Thermo Fisher Scientific, MA, U.S.A, RRID:SCR_018042).

Total RNA libraries were prepared for transcriptome sequencing using the Zymo-Seq RiboFree Stranded Total RNA library preparation kit (Zymo Research, CA, U.S.A) following the manufacturer’s instructions. Further, rRNAs were depleted as part of the sample preparation process. Libraries were validated for length and adapter dimer removal using the Agilent TapeStation 4200 D1000 High Sensitivity assay (Agilent Technologies, CA, U.S.A, RRID:SCR_019398). Subsequent quantification and normalization were carried out using the dsDNA High Sensitivity Assay for Qubit 3.0 (Life Technologies, CA, U.S.A, RRID:SCR_020311). Sample libraries were then prepared for Illumina sequencing following the manufacturer’s protocol (Illumina, CA, U.S.A). All the individual samples were consolidated into a single sequencing pool, with proportions targeting 30M reads/sample, and sequenced on the Illumina NovaSeq 6000 platform (Illumina, CA, U.S.A, RRID:SCR_016387).

Raw reads were subjected to adaptor removal and quality control including filtering of low-quality reads. Filtered reads were mapped to the mouse reference genome using STAR (Spliced Transcripts Alignment to a Reference, RRID:SCR_004463). Gene expression level was estimated by transcripts per million of transcript sequences. Principal component analysis (PCA) was performed to visualize overall gene expression differences between compared groups in several major principal components. Differential gene expression was analyzed by DeSeq2 (RRID:SCR_015687) analysis package. Significantly regulated genes were presented with different cutoffs of adjusted p values. We utilized Ingenuity Pathway Analysis (IPA, RRID:SCR_008653) to predict pathways associated with differentially expressed genes and employed Gene Set Enrichment Analysis (GSEA, RRID:SCR_003199) to visualize the outcomes in a heatmap.

### 16S rRNA sequencing and microbiome data analysis

Microbial genomic DNA extraction from mouse stool samples was carried out using the Quick-DNA Fecal/Soil Microbe 96 Kit (Zymo Research, CA, U.S.A) following the manufacturer’s protocols. The hypervariable region V4 of the bacterial 16S rRNA gene, 515F (5’- GTGYCAGCMGCCGCGGTAA-3’) and 806R (5’- GGACTACNVGGGTWTCTAAT-3’) was amplified and sequenced on the Illumina MiSeq platform (2×250bp) (Illumina, CA, U.S.A). The raw sequencing reads were processed via the DADA2 V1.16 (RRID:SCR_023519) data processing pipeline to generate amplicon sequence variants (ASVs). Taxonomic assignments were made using the Silva database V138.1 (RRID:SCR_006423), with a classification confidence threshold set at *p* < 0.5 for assigning unclassified taxa at their respective taxonomical levels. PCR negative controls and extraction controls for DNA extraction and sequencing were included in the analysis. Notably, all negative controls yielded fewer than 500 reads, indicating that background noise minimally impacted the data analysis. Sequencing reads from all the samples were rarified to 10,000 reads per sample. All of the microbiome analysis was done in R.

### General statistical approaches

Statistical analysis and data mining were conducted using GraphPad Prism 8 (GraphPad, CA, USA, RRID:SCR_002798). For the calculation of ethanol consumption during the DID procedure, it was expressed as grams of ethanol per kilogram of body weight (g/kg body weight), where 20% ethanol intake was determined as follows: multiplying the drinking volume by the ethanol percentage within that volume and the density of ethanol, and then dividing this by the mouse’s body weight in kilograms. To assess the effects of antibiotic cocktail administration on ethanol consumption and BEC between the Abx and PBS group at both baseline and post-Abx treatment time points, a paired t-test was employed between groups. An analysis of variance (ANOVA) was used for comparing various SCFA levels before and after Abx administration. Furthermore, the impact of all SCFA supplementation on ethanol consumption was evaluated using the ANOVA. The food intake was assessed by providing a pre-weighed amount of food in their cage hopper and then weighing the remaining food inside the cage and in the hopper daily. To assess daily fluid intake, the sodium valerate and NaCl bottles were pre-weighed, and the remaining amount was deducted after the measurement period. Lastly, intakes were normalized to mouse body weight by dividing the average intake by the average body weight. The GABA and histone acetylation differences between groups were assessed by the Mann-Whitney test. For the analysis of anxiety-like behavior, one-sample t-tests were employed to examine the behavioral parameters following sodium valerate supplementation. Overall microbiome differences between groups were visualized by PCA. Relative abundance difference of specific taxa was identified using Wilcoxon-Sum Rank test or Linear discriminant analysis Effect Size (LefSe, RRID:SCR_014609) analysis. Gut-brain modules were calculated using the R version of the Gomixer tool. Right tailed Fisher’s exact test was performed in the IPA predicted canonical pathways for statistical analysis.

## Results

### Antibiotic administration increases ethanol consumption but reduces fecal SCFA levels

**Figure 1:**
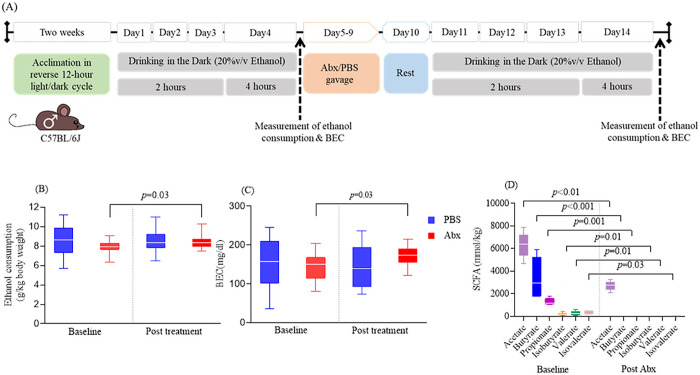
Effect of oral antibiotics on ethanol consumption, BEC and levels of SCFA in stool (A) A schematic depiction illustrating the DID paradigm and Abx treatment. The gray horizontal bar represents the 20% v/v ethanol drinking for two hours during the first three days and for four hours on the fourth day. The light orange horizontal bar symbolizes administration antibiotics (Abx) or PBS by oral gavage. The light blue horizontal bar represents the rest day post antibiotic treatment. The dashed black arrow represents the measurement of ethanol consumption and BEC at the completion of the 4-hour ethanol drinking. (B) Ethanol consumption normalized by body weight at end of 4hr drinking period (n=14 mice/group). (C) BEC at end of 4hr drinking period (n=14 mice/group). (D) Effect of Abx administration on SCFA levels in stool (n=7 mice). In panel B and C of the figure, a paired t-test was used to compare two groups, while in panel D, one way ANOVA was employed to compare SCFA levels before and after Abx administration. In all three panels (B-D), the data are presented with medians (−), 25th and 75th percentiles (box), maximum and minimum values (whiskers). The significant *p*-values are presented within the figure.

To evaluate the contribution of the overall microbiome community on ethanol consumption, we measured ethanol consumption (unit: g/kg body weight) and BEC (unit: mg/dl) in adult male mice (n = 14 mice/group) using a DID paradigm at baseline and after Abx treatment. A schematic diagram illustrating the administration of Abx and the DID procedure is depicted in Fig. 1A. At baseline, no statistically significant differences in ethanol consumption (Fig. 1B) or BEC were noted (Fig. 1C). Following a 10-day treatment period with Abx, we once again employed the DID paradigm in both the Abx-treated mice and PBS control mice. Notably, after this treatment period, the Abx-treated mice exhibited a significantly higher level of ethanol consumption (*p* = 0.03; Fig. 1B) and BEC (*p* = 0.03; Fig. 1C) compared to their baseline levels. In contrast, the PBS control mice did not display a significant difference in either consumption or BEC when compared to their baseline levels.

In mice, SCFA levels have been implicated in modulating alcohol consumption [20, 22] and in principle, oral antibiotics treatment suppresses the levels of intestinal SCFA [28, 29]. To investigate whether levels of SCFAs are altered in Abx treated mice, we analyzed the SCFA at baseline and after Abx treatment in the mice stool. At baseline, SCFAs such as acetate, butyrate, and propionate are usually found at high concentrations, while isobutyrate, valerate, and isovalerate are found at lower concentrations (n = 7 mice). All SCFAs were abolished after antibiotic treatment (*p* < 0.05), with only minimal levels of acetate (mean ± SD = 2722.21 ± 372.26 mmol/kg stool) and isovalerate (mean ± SD = 23.8 ± 17.02 mmol/kg stool) remaining (Fig. 1D). Together, these observations demonstrate that oral antibiotic administration increases alcohol consumption and decreases the levels of fecal SCFAs.

### Sodium valerate supplementation reduces ethanol consumption, BECs, and anxiogenic behavior

**Figure 2:**
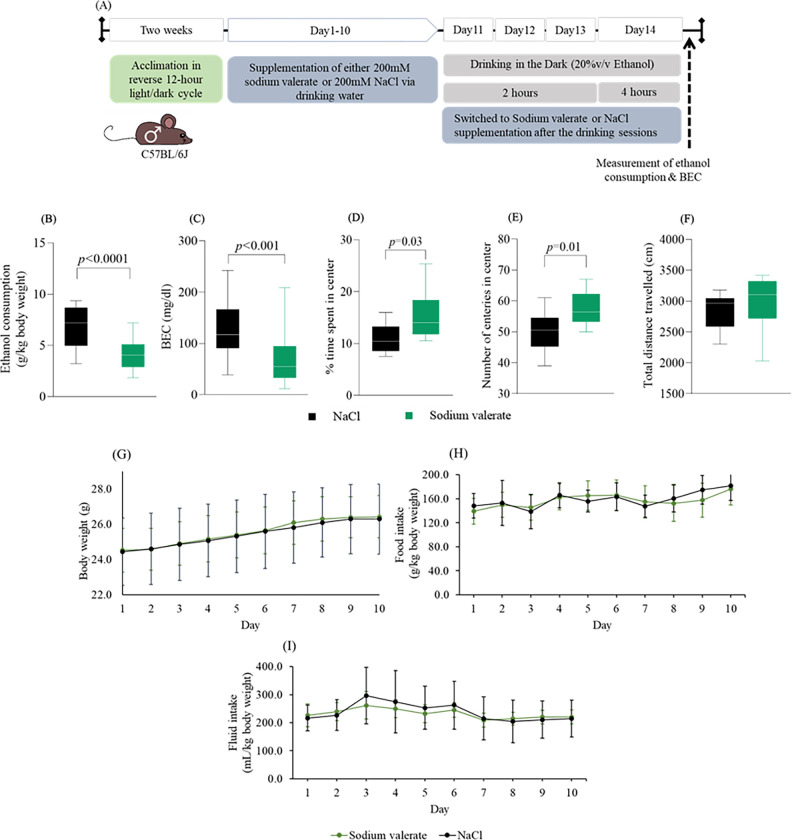
Effect of sodium valerate on ethanol consumption, BEC, anxiety and diet (A) This schematic illustrates incorporation of sodium valerate and NaCl supplementation into the DID paradigm. The gray horizontal bar represent the 20% v/v ethanol drinking for two hours during the first three days and for 4 hours on the fourth day within the DID paradigm. The blue-gray horizontal bar represents either sodium valerate in sodium valerate supplemented mice or NaCl supplementation in control mice. This supplementation continued throughout the entire experiment, except during DID sessions. The dashed black arrow indicates the measurement of ethanol consumption and BEC through blood collection after the completion of the 4-hour ethanol drinking session. (B) Ethanol consumption, and (C) BEC in mice supplemented either with sodium valerate or NaCl (n=21 mice/group). (D) Percentage of time spent in the center, (E) number of center entries, and (F) distance travelled in the center area during the open field test of mice supplemented with sodium valerate or NaCl (n=10 mice/group). In panel B and C of the figure, a Mann-Whitnay test was used to compare two groups, while in panel D-F, one sample t-test was employed to compare behavior parameters between two groups. In each of the four panels (B-F), the data are depicted using medians (−), 25th and 75th percentiles (box) and maximum and minimum values (whiskers). (G) Daily body weight, (H) food consumption and (I) intake of sodium valerate and NaCl in respective groups over a 10-day period. Each line represents mean ± SD for each group. The significant *p*-values are presented within the figure.

Prior studies reported that SCFA supplementation has promise as a potential strategy to reduce ethanol intake and preference [22]. To further explore this relationship, we investigated the effect of several SCFAs (sodium butyrate, acetate and valerate) supplementation (oral gavage, once daily for 10 days) on ethanol consumption in mice (n = 7 mice/group) via the DID paradigm. Notably, sodium valerate supplemented mice consumed less ethanol than both NaCl and other SCFA supplemented mice. A significant difference (*p* = 0.004) was observed between sodium valerate and sodium butyrate supplemented mice (**Supplementary figure A-B**). We repeated the sodium valerate supplementation study in a larger cohort of male mice (n = 21 mice/group) (Fig. 2A). The effect of sodium valerate supplementation on reducing ethanol consumption (*p* < 0.0001; Fig. 2B) and BEC (*p* < 0.001; Fig. 2C) was significant. Sodium valerate supplementation led to a 40% reduction in ethanol consumption, with a median of 4.03 g/kg body weight compared to 7.16 g/kg consumed body weight in the NaCl supplemented mice. Additionally, BEC was lowered by 53% in sodium valerate-supplemented mice, with a median of 54.3 mg/dl compared to a median of 116.3 mg/dl in NaCl supplemented control mice.

Studies have shown that administering SCFAs have potential anxiolytic effects. We examined the effects of sodium valerate and NaCl on anxiety-like behavior (n = 10 mice/group) after 10 days of supplementation by open-field activity test. As shown in Fig. 2D–E, sodium valerate supplementation produced a significant increase in percentage of time spent in center are [(mean ± SD = 15.29 ± 4.82; *p* = 0.03) and number of entries in center [(mean ± SD) = 57.50 ± 5.48; *p* = 0.01] during a 10 min exploration period. However, it did not lead to a significant change in the distance travelled (Fig. 2F). Overall, supplementation of sodium valerate reduces anxiety-like or approach-avoidance behavior in mice.

Although the mice gained weight throughout the study, the average weight did not differ significantly (*p* > 0.05; n = 10/group) between the groups (Fig. 2G). In addition, we observed no statistical difference (*p* > 0.05; n = 14/group) in food consumption or intake of sodium valerate and NaCl among the respective groups (Fig. 2H and 2I).

### Enhanced GABA levels following sodium valerate supplementation

**Figure 3:**
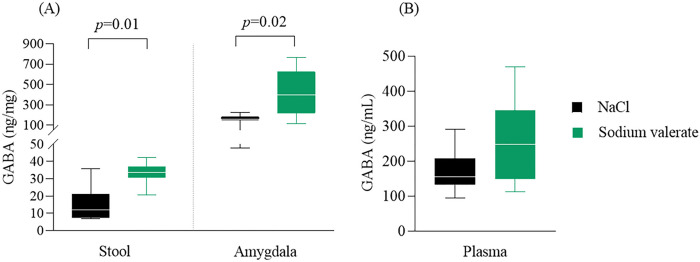
Effect of sodium valerate supplementation on GABA levels. (A) Concentrations of GABA in stool and amygdala, and (B) concentrations of GABA in plasma of sodium valerate and NaCl control mice after 10 days of supplementation following the DID paradigm (n= 7 mice/group). The data are illustrated with medians (−), the 25th and 75th percentiles (box), and the range between maximum and minimum values (whiskers). The Mann-Whitney test was utilized to compare two groups within each sample type. The significant *p*-values are provided within the figure.

Research has shown that changes in the neurotransmitter GABA can contribute to the development of alcohol dependence [30] and anxiety-like behaviors [31] and valeric acid shares similar structural features with the neurotransmitter GABA [25]. To understand the impact of valeric acid on GABA levels, we performed an ELISA assay in different samples, including plasma, stool, and the amygdala (n = 7 mice/group). We found that sodium valerate supplementation led to elevated GABA levels in stool (*p* < 0.01) and the amygdala (*p* = 0.03), compared to the control group supplemented with NaCl (Fig. 3A). A similar trend was also evident in plasma (Fig. 3B) but was not significant (*p* = 0.17). No detectable levels were observed when directly testing valeric acid with the ELISA assay. This result suggests a potential connection between valeric acid supplementation, heightened GABA levels, and a reduction in alcohol consumption.

### Sodium valerate supplementation increases histone acetylation in the amygdala

**Figure 4:**
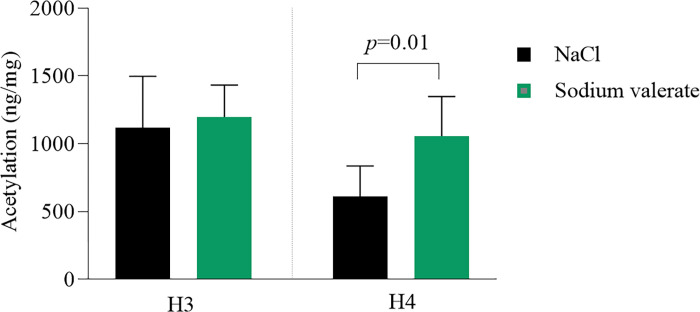
Effect of sodium valerate supplementation on levels of histone acetylation. Acetylation of H3 and H4 in the amygdala of sodium valerate and NaCl control mice after 10 days of supplementation following the DID paradigm (n= 7 mice/group). Each bar represents the mean acetylation ± SD of respective groups. The Mann-Whitney test was utilized to compare two groups and the significant *p*-value is mentioned within the figure.

Decreased histone acetylation was reported as a consequence of binge drinking. SCFAs are well-known histone deacetylase inhibitors. To determine the effect of sodium valerate on histone acetylation, we measured the acetylation levels of H3 and H4 in the amygdala following 10 days of sodium valerate supplementation (n = 14 mice/group). We found H4 hyperacetylation significantly increased in mice supplemented with sodium valerate compared to NaCl controls (*p* = 0.01), while the observed increase in H3 acetylation with sodium valerate was not statistically significant (*p* = 0.71) in the amygdala of these mice (Fig. 4). These results suggest that dietary supplementation with sodium valerate may contribute to the increased acetylation of H4 in the amygdala, potentially contributing to a decrease in ethanol consumption.

### Amygdala transcriptome analysis in mice supplemented with sodium valerate

**Figure 5:**
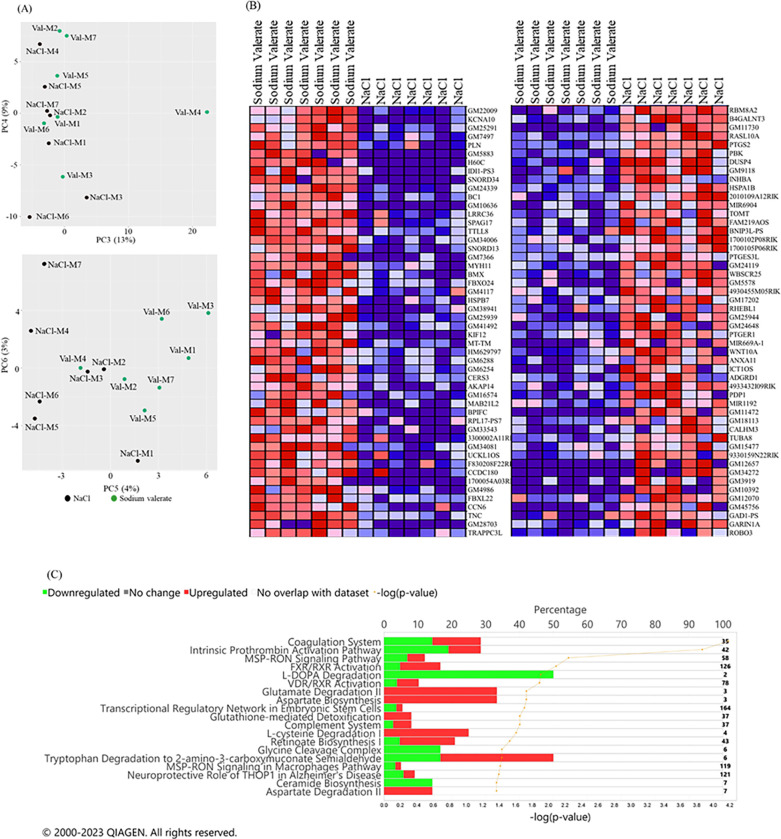
Effect of sodium valerate on gene expression in the amygdala. (A) PCA plot illustrates sample characteristics from both groups based on gene expression levels, with each dot representing a sample (B) The heatmap displays the top 50 upregulated and top 50 downregulated DEGs in mice supplemented with sodium valerate (left column) and NaCl (right column). The expression values are depicted as ranging from red (high expression) to pink (moderate), light blue (low), and dark blue (lowest expression). (C) Highlights some of the top canonical pathways identified by IPA that are affected by sodium valerate supplementation. The representation of pathway regulation is expressed as a percentage (top axis) using bar graphs, with respective *p*-values (bottom axis) derived from right-tailed Fisher’s exact test represented by yellow line. Here n=7 mice/group were used for gene expression analysis.

To gain a molecular understanding of the effects of sodium valerate supplementation on brain function, we performed bulk RNA sequencing (RNA-seq) analysis on amygdala tissue of mice that underwent the DID paradigm after sodium valerate or NaCl (n = 7 mice/group) supplementation. Principal component analysis (PCA) showed no clear distinction between sodium valerate supplemented mice and NaCl supplemented mice at PC1 and PC2 components, but a clear separation in PC3 to PC6 (Fig. 5A), suggesting sodium valerate supplementation has a marked influence on gene expression in the amygdala. Differential Expression analysis of RNA-Seq 2 (DESeq2) identified a total of 301 differentially expressed genes (DEG) at the adjusted *p* value < 0.25, 126 genes at the adjusted *p* value < 0.10, and 75 genes at the adjusted *p* value < 0.05. Among the 75 significant DEG, 47 genes showed upregulation, while 28 displayed downregulation. The heatmap displays the top 50 genes in the sodium valerate versus NaCl control (Fig. 5B). Among these genes, the top five protein coding genes with the highest expression in sodium valerate-supplemented mice are *Kcna10* (potassium voltage-gated channel, shaker-related subfamily, member 10), *Pln* (phospholamban), *H60C* (histocompatibility antigen 60c), *Idi1* (isopentenyl-diphosphate delta isomerase, pseudogene 3) and *Snord34* (small nucleolar RNA, C/D box 34). The top five most strongly down-regulated genes in the sodium valerate group are *Rbm8a2* (RNA binding motif protein 8A2), *B4galnt3* (beta-1,4-N-acetyl-galactosaminyl transferase 3), *Rasl10a* (RAS Like Family 10 Member A), *Ptgs2* (prostaglandin-endoperoxide synthase 2) and *Pbk* (PDZ binding kinase). *PTGS2* is a prostanoids producer that responds to inflammation. Its downregulation in the sodium valerate supplemented group (log_2_Fold change: −0.65; adjusted *p* = 0.0002) indicates a potential anti-inflammatory effect of valerate. Mitogen-activated protein kinases (MAPKs), a group of protein kinases that regulate critical inflammatory processes are also downregulated at the RNA level in the sodium valerate supplemented group. As SCFAs may function in the brain through G protein-coupled receptors (GPCRs), we examined gene expression levels of 85 GPRs in the RNAseq data. We found *Gpr56* and *Gpr158* are highly expressed among all these GPCRs. *Gpr56*, whose expression has been associated with antidepressant response, was upregulated in sodium valerate supplemented mice (log_2_Fold change: 0.28; unadjusted *p* = 0 < 0.01). Conversely, *GPR158* is associated with depression after chronic stress, and is downregulated in the sodium valerate supplemented group (log_2_Fold change: −0.26; unadjusted *p* < 0.01). Notably, we found that six mitochondrial genes encoding subunits of the enzyme NADH dehydrogenase (Complex I) were consistently upregulated in the sodium valerate supplemented group.

IPA was performed to identify changes to the molecular signaling pathways and to comparatively assess the sodium valerate effects in the amygdala. The regulated pathways predicted in sodium valerate supplemented mice are associated with downregulation of L-dopa degradation, upregulation of aspartate biosynthesis, glutamate degradation, tryptophane degradation, the bidirectional regulation of pulmonary blood coagulation, retinoate biosynthesis, complement system, FXR/RXR activation, LXR/RXR activation, as well as the upregulation of L-cysteine degradation and the downregulation of glycine cleavage complex ceramide biosynthesis, etc. (Fig. 5C). Thus, sodium valerate treatment induces significant changes in gene expression spanning various signaling processes including neuroinflammation, neurotransmission, mitochondria regulation, and GPCR signaling.

### Sodium valerate supplementation shifts the gut microbiota community and gut-brain modules

**Figure 6:**
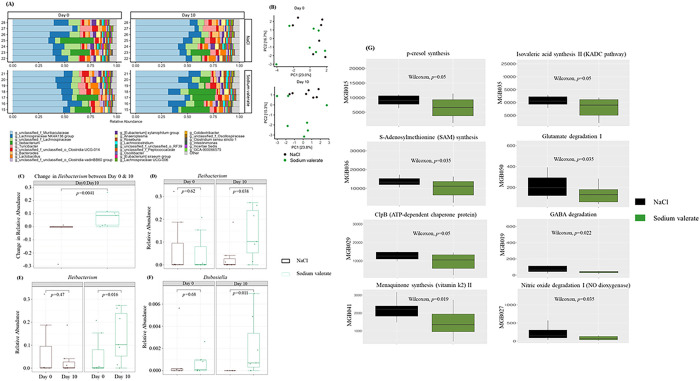
Gut microbiome, gut-metabolites and gut-brain modules in sodium valerate supplemented mice A) Stacked bar plot illustrates the percentage of top 25 genera present in stool samples from sodium valerate and NaCl supplemented mice as identified by sequencing of V4 16S amplicons. (B) PCA plots showcase the variation in beta-diversity between mice supplemented with NaCl and sodium valerate at Day 0 and Day 10. Each dot represents a sample from an individual mouse. (C) Displays of the changes of relative abundance of *Ileibacterium* after the 10-day period of sodium valerate and NaCl supplementation from the baseline day 0. (D) Shows of the differences in relative abundance of *Ileibacterium* between the groups at day 0 and day 10. (E) Highlights of the variations in relative abundance of *Ileibacterium* within each group at day 0 and day 10. (F) Demonstration of the differences in relative abundance of *Dubosiella*between the groups at day 0 and day 10. In panels C-G, the data are presented using medians (−), the 25th and 75th percentiles (box), the range between maximum and minimum values (whiskers), and individual data points, including outliers. (G) Depiction of predicted gut-brain modules in mice supplemented with sodium valerate and NaCl. Relative abundance difference of specific taxa was identified using Wilcoxon-Sum Rank test or LefSe. Here n=7 mice/group were used for gene expression analysis.

SCFAs are products of gut microbiota metabolism and can also regulate the composition of the gut [32]. To determine if sodium valerate supplementation affects the gut microbiome community composition, we performed 16S rRNA gene sequencing of stool samples before and after sodium valerate treatment. The most abundant genera were unclassified *Muribaculaceae, Lachnospiraceae* NK4A136 group, and unclassified *Lachnospiraceae* (Fig. 6A). Permutational multivariate analysis of variance (PERMANOVA) tests were performed on CLR-transformed data to test for beta-diversity differences between sodium valerate and NaCl-supplemented groups (n = 14 mice/group). Prior to any supplementation at day 0, no significant difference was observed between the groups (*p* = 0.692). However, after 10 days of supplementation, the groups showed increased dissimilarity (*p* = 0.067) and exhibited distinct clustering patterns when visualized in a PCA plot, indicating a supplementation-related impact on the composition of the gut microbiome (Fig. 6B). Among all the genera analyzed, an increase in abundance was noted only in the *Ileibacterium* genera in sodium valerate supplemented mice (adjusted *p* = 0.24, unadjusted *p* = 0.0041) (Fig. 6C). This finding was further confirmed via Wilcox tests on the relative abundance at day 10 between sodium valerate and NaCl mice (unadjusted p = 0.038) (Fig. 6D), and the difference in abundance between day 0 and day 10 for the sodium valerate mice (unadjusted p = 0.011), which supported an increase in *Ileibacterium* in sodium valerate supplemented mice (Fig. 6E). Further analysis with DESeq2 found that the genus *Dubosiella* was significantly (log_2_Fold change = 7.84, adjusted *p* = 9.46E-5) more abundant in mice after 10 days of sodium valerate supplementation (Fig. 6F). This finding was confirmed with a Wilcox test (unadjusted *p* = 0.011).

To determine neuroactive potential of the valerate-altered gut microbiota, we inferred neuroactive compound production and degradation process based on gut-brain module (GBM) analysis. Thirty-four GBMs were identified in the collected samples, 8 GBMs exhibited significant differences (*p* < 0.05) between the sodium valerate and NaCl supplemented mice. Interestingly, all 8 GBMs showed a significant decrease in valerate treated mice (Fig. 6G), including p-Cresol synthesis, Isovaleric acid synthesis II, S-Adenosylmethionine synthesis, Glutamate degradation I, GABA degradation, Nitric oxide degradation I, ClpB (ATP-dependent chaperone protein), and Menaquinone synthesis (vitamin K2) II. This data suggests that sodium valerate supplementation prevents GABA degradation and increases its availability through modulation of the gut microbiome.

## Discussion

Our study discovered that a sodium salt of gut microbial metabolite valeric acid reduces binge-like alcohol consumption in mice. This effect is associated with an increase of GABA levels in the periphery and brain, modulation of brain epigenetics and transcriptomics, and an impact on the gut microbiome composition.

Previous research has demonstrated the involvement of gut microbiota in alcohol consumption, [8–10] and the disruption of gut microbiota through antibiotics has been shown to increase ethanol consumption in mice [33]. In agreement with this study, we found that Abx treatment significantly increased voluntary ethanol consumption levels in a binge-like ethanol drinking paradigm in mice. However, the opposite effect was reported in wistar-derived high-drinker UChB rats [34], it’s worth noting that it utilized an ad libitum ethanol access paradigm leading to lower BEC. In addition, a different antibiotics regimen, and animals were also used in their study. Regardless, our work reveals an important relationship between the gut microbiota and ethanol consumption behavior and supports the use of microbial-targeted approaches to study gut-brain interactions in alcohol drinking behavior.

In our mouse model, intestinal SCFA production was significantly suppressed by the Abx treatment. This finding is consistent with previous reports. Additionally, in our study where we provided various SCFAs to mice as supplements, we observed no statistical changes in alcohol intake when sodium acetate and butyrate were supplemented. Prior research suggests that acetate might encourage heavy drinking, providing a reward in the form of added energy from calories or by influencing adenosinergic adaptation mechanisms [22]. Studies have shown that sodium butyrate does not influence alcohol self-administration in non-dependent rodents but may reduce drinking in alcohol-dependent or antibiotic treated rodents. Interestingly, when we supplemented valeric acid, we observed a significant reduction in ethanol consumption. Valeric acid can also be found in plants such as *Valeriana wallichii* and *Valeriana officinalis*. One study has shown that *Valeriana wallichii* extract reduces chronic ethanol intake in animal models. However, *Valeriana wallichii* extract contains a variety of active constituents. The exact compound that is responsible for reduced ethanol intake has not been studied. In our study, sodium valerate supplementation did not affect body weight, food intake, or fluid drinking. This suggests that the observed reduction in alcohol consumption was not due to changes in fluid or weight regulation. A recent study reported a decrease in fecal isovalerate (an isomer of valeric acid) linked to increased alcohol drinking in humans, which further reiterates the potential of valeric acid in regulating ethanol consumption.

The molecular mechanisms that underlie alcohol drinking behaviors are intricate and multifaceted [35]. Anxiety can promote alcohol drinking behaviors in both humans and animals, and excessive drinking increase anxiety-like behavior [36–38]. Our study suggests that sodium valerate supplementation have potential anxiolytic effects in mice. Interestingly, *Valeriana wallichii* and *Valeriana officinalis*, plant reservoirs of valeric acid and other compounds, have been used as supplements to address insomnia and anxiety due to their sedative attributes [39–41]. GABA may plays a role in reducing depression and anxiety linked to alcohol dependence, as lower GABA levels are associated with these conditions [42, 43]. In our study, sodium valerate supplement led to increased GABA levels in stool and the amygdala. Valproic acid, a structural analog of valeric acid, is a popular antiepileptic drug with GABAergic activity. Some research suggests that valproic acid may raise GABA levels in the brain by inactivating α-ketoglutarate dehydrogenase involved in the breakdown of GABA [44]. Whether valeric acid acts in a similar fashion warrants further investigation.

Increased levels of GABA detected in stool samples from mice supplemented with sodium valerate suggest that the gut microbiome may be involved in GABA regulation. Previous studies have identified a variety of GABA-producers and degraders in the gut microbiome. Indeed, our gut-brain module analysis revealed a decrease in GABA degradation by the gut microbiome in sodium valerate supplemented mice. It will be interesting to examine the ability of GABA modulation by *Ileibacterium* and *Dubosiella*, two bacterial genera that are significantly increased during sodium valerate supplementation. However, it is also possible that changes in *Ileibacterium* and *Dubosiella* abundance are responses to administered sodium valerate or increased GABA. A report indicated that the metabolic disorder induced by chronic alcohol consumption caused a decrease in the relative abundance of *Ileibacterium*. Under physiological conditions, it has been widely believed that GABA does not cross blood brain barrier [45, 46]. The impact of gut-derived GABA on brain function and drinking behavior, therefore, warrants further investigation. It is also possible that valeric acid can directly cross blood brain barrier and regulates GABA levels in the brain or acts indirectly through gut-brain axis.

There is consistent evidence that acute and chronic alcohol exposure modulate histone acetylation in the amygdaloid circuitry, leading to alcohol tolerance and dependence. Our study revealed increased acetylation of histone H4 in the amygdala of sodium valerate-supplemented mice. Previous findings confirm that intermittent alcohol exposure decreased histone acetylation in the amygdala, which may be related to the ethanol-induced increase in histone deacetylase (HDAC) [47]. Similarly, administration of HDAC inhibitors like sodium butyrate increase histone acetylation and suppress anxiety or depression-like behaviors in mice. Our results suggest that HDAC inhibitors such as sodium valerate may be able to reverse the effects of ethanol via HDAC-induced epigenetic changes in the amygdala.

Increased histone acetylation leads to a more open structure of chromosomes, thus promoting gene transcription. Our data suggests that another potential mechanism by which valeric acid attenuates alcohol drinking is through its effects on transcriptional regulation in the brain. A downregulation of inflammatory molecules such as *Ptgs2* and *MAPK* was identified in valerate-treated mice. The immune modulatory effect of valerate has been shown in experimental mouse models of colitis and multiple sclerosis, mediated by suppressing Th_17_ cells and the enhancement of IL-10 production [48]. Numerous studies have demonstrated that SCFAs modulate transcription of a wide range of genes associated with behaviors [19]. SCFAs are known to regulate GPCRs such as *GPR41, GPR43*, and *GPR109A*, all of which are critical in regulating neuroinflammation, depression, and anxiety-like behaviors [49]. Our bulk RNAseq analysis showed upregulation of *GPR56* and downregulation of *GPR158* in the amygdala region of the brain in valerate treated mice. *GPR158* is a novel regulator of stress-responsive behaviors and is highly upregulated in people with major depression disorder [50]. By contrast, *GPR 56* activation has an antidepressant effect [51]. Valerate acid may regulate two GPRs of opposing effects to control anxiety or depression behavior, thus indirectly influencing moderating drinking behavior.

Our study has some limitations. All experiments in the current study were performed in male mice, further investigation is necessary to ascertain if there also exists an association between sodium valerate and alcohol intake in females. The study was tested on alcohol-independent mice. Future studies will be conducted to assess treatment effects of sodium valerate supplementation on alcohol-dependent animals. Further research is needed to fully understand the underlying mechanisms of action of valerate acid on voluntary alcohol drinking.

## Conclusion

Sodium valerate supplementation shows promise as a novel intervention to reduce alcohol consumption. This effect potentially acts through the modulation of multiple molecular targets associated with the pathogenesis of excessive alcohol use. Our study contributes to the growing understanding of the gut-brain axis and provides insights into potential therapeutic strategies for excessive alcohol consumption and related anxiety.

## Data Availability

Upon a reasonable request, all datasets will be available from the corresponding author.

## Abbreviations

Abx: antibiotic cocktail
BEC: blood ethanol concentration
DEG: differentially expressed genes
DeSeq2: differential expression analysis of RNA-Seq 2
DID: drinking in the dark
GABA: γ-aminobutyric acid
GPCR: G protein-coupled receptors
HDAC: histone deacetylase
IPA: ingenuity pathway analysis
NaCl: sodium chloride
MAPK: mitogen-activated protein kinases
PBS: phosphate-buffered saline
PCA: principal component analysis
SCFA: short-chain fatty acid
